# Exosome-like nanoparticles derived from fruits, vegetables, and herbs: innovative strategies of therapeutic and drug delivery

**DOI:** 10.7150/thno.97096

**Published:** 2024-08-01

**Authors:** Bo Zhao, Hangjuan Lin, Xinchi Jiang, Wanshu Li, Yuli Gao, Minghui Li, Yanan Yu, Ninggang Chen, Jianqing Gao

**Affiliations:** 1 Department of Pharmacy, Ningbo Municipal Hospital of Traditional Chinese Medicine (TCM), Affiliated Hospital of Zhejiang Chinese Medical University, Ningbo 315016, China.; 2 State Key Laboratory of Advanced Drug Delivery and Release Systems, College of Pharmaceutical Sciences, Zhejiang University, Hangzhou 310058, China.; 3 Department of Dermatology Medical Cosmetology Center, Ningbo Municipal Hospital of Traditional Chinese Medicine (TCM), Affiliated Hospital of Zhejiang Chinese Medical University, Ningbo 315016, China.

**Keywords:** exosomes, plant-derived exosome-like nanoparticles, biotherapy, drug delivery, challenge

## Abstract

Over the past ten years, significant advancements have been made in exploring plant-derived exosome-like nanoparticles (PELNs) for disease therapeutics and drug delivery. PELNs, as inherent nanoscale particles comprised of proteins, lipids, nucleic acids, and secondary metabolites, exhibit the capacity for cellular uptake by human cells. This intercellular interaction transcends biological boundaries, effectively influencing biological functions in animals. PELNs have outstanding biocompatibility, low immunogenicity, enhanced safety, and environmentally friendly sustainability. This article summarized the preparation methods and characteristics of PELNs. It provided a systematic review of the varied roles of PELNs derived from fruits, vegetables, and herbs in disease therapeutics and drug delivery. The challenges in their production and application were discussed, and future prospects in this rapidly evolving field were explored.

## 1. Introduction

There have been significant advancements over the past decade in therapeutic strategies of cell-derived products, particularly extracellular vesicles (EVs). Exosomes, the smallest and most extensively studied EVs, can be produced by bacteria, animals, and plants 1 to facilitate intercellular communication 2. There is accumulating evidence that exosomes are involved in the progression of diverse disease processes and possess multifaceted biological functions, such as antigen presentation, modulation of immune responses, and cytokine transport 3-5. Due to their favorable biocompatibility, low cytotoxicity, and minimal immunogenicity, exosomes have been applied in the diagnosis and treatment of various diseases 6. Furthermore, exosomes exhibit specific targeting capabilities and the ability to traverse biological barriers, positioning them as promising carriers for drug delivery.

Traditionally, research has primarily focused on exosomes derived from stem cells and tumor cells but with advancements in nanoscience, increasing attention is being directed towards the presence and role of exosomes in edible plants. The exploration of plant exosomes commenced when Regente et al. first isolated exosomes in plants (sunflower seedlings) through transmission electron microscopy and proteomic analysis in 2009 7. However, there is a lack of consensus definitions, consistent nomenclature, and standardized practices for the extracted biological vesicles derived from plants owing to the lack of well-defined biological pathways and rigorous physicochemical characterization. These nanoparticles have been termed plant-derived exosome-like nanoparticles (PELNs) in this review. Their therapeutic potential has been examined, revealing structural, cargo, and release mechanisms similar to exosomes from animal sources 8. Particularly, microRNAs (miRNAs) from plants discovered in the serum of healthy Chinese males and females were found to regulate the translation of mammalian LDLRAP1 similarly to mammalian miRNAs 9. Therefore, miRNAs derived from plants may exert biological effects across different species. It is noteworthy that a multitude of miRNAs have been detected in PELNs, which are absorbed by the human body and exert therapeutic effects 10-13. PELNs could potentially serve as a pathway for plants to transfer miRNAs to animals.

PELNs have garnered significant attention due to their abundant sources and broad potential applications in biomedical and nanotechnology fields. This article categorized plants into three groups: fruits, vegetables, and herbs, and explored the biomedical applications of PELNs derived from each group. Fruits, such as grapes, lemons, and apples, are commonly consumed as desserts or snacks. Vegetables, including ginger, broccoli, and bitter melon, are predominantly used in cooking or salads. Herbs, such as ginseng, pueraria lobata, and lonicera japonica, are typically used for medicinal purposes. PELNs derived from these sources exhibit therapeutic functions such as anti-inflammatory, anti-tumor, and gut microbiota modulation properties. Fruit-derived PELNs demonstrate antioxidant capabilities, vegetable-derived PELNs show antiviral and insulin resistance regulation abilities, and herb-derived PELNs have regenerative and anti-osteoporosis effects. Furthermore, PELNs have been applied as drug delivery carriers owing to their exceptional biocompatibility, stability, and ability to deliver both hydrophilic and hydrophobic cargo in various therapeutic applications, such as tumor-targeted delivery, colon-targeted delivery, transdermal delivery, and gene delivery 14-17. This article provides an overview of the biogenesis, composition, and uptake mechanisms of PELNs to solidify the substantial potential of PELNs derived from fruits, vegetables, and herbs in biotherapy and drug delivery while highlighting the limitations and challenges associated with their applications.

## 2. Overview of PELNs

Plant cells release PELNs in response to various environmental stresses such as pathogenic infections and these PELNs share similarities with exosomes derived from animal sources in terms of surface charge, morphology, and content composition 18. PELNs contain miRNA, lipids, and proteins, serving as extracellular messengers that mediate intercellular communication. Moreover, PELNs have an inherent capability to deliver chemical molecules. The significance of PELNs in inter-species communication arises from their diversity of biomolecules (proteins, lipids, nucleic acids, and secondary metabolites) and their ease of internalization by mammalian cells 19. Hence, PELNs present a promising avenue for the efficient delivery of targeted drugs, offering a natural therapeutic strategy for a wide range of diseases. Furthermore, unlike exosomes derived from mammals, PELNs provide an abundant and renewable source that allows for large-scale production 20.

### 2.1 Biogenesis of PELNs

**Figure 1:**
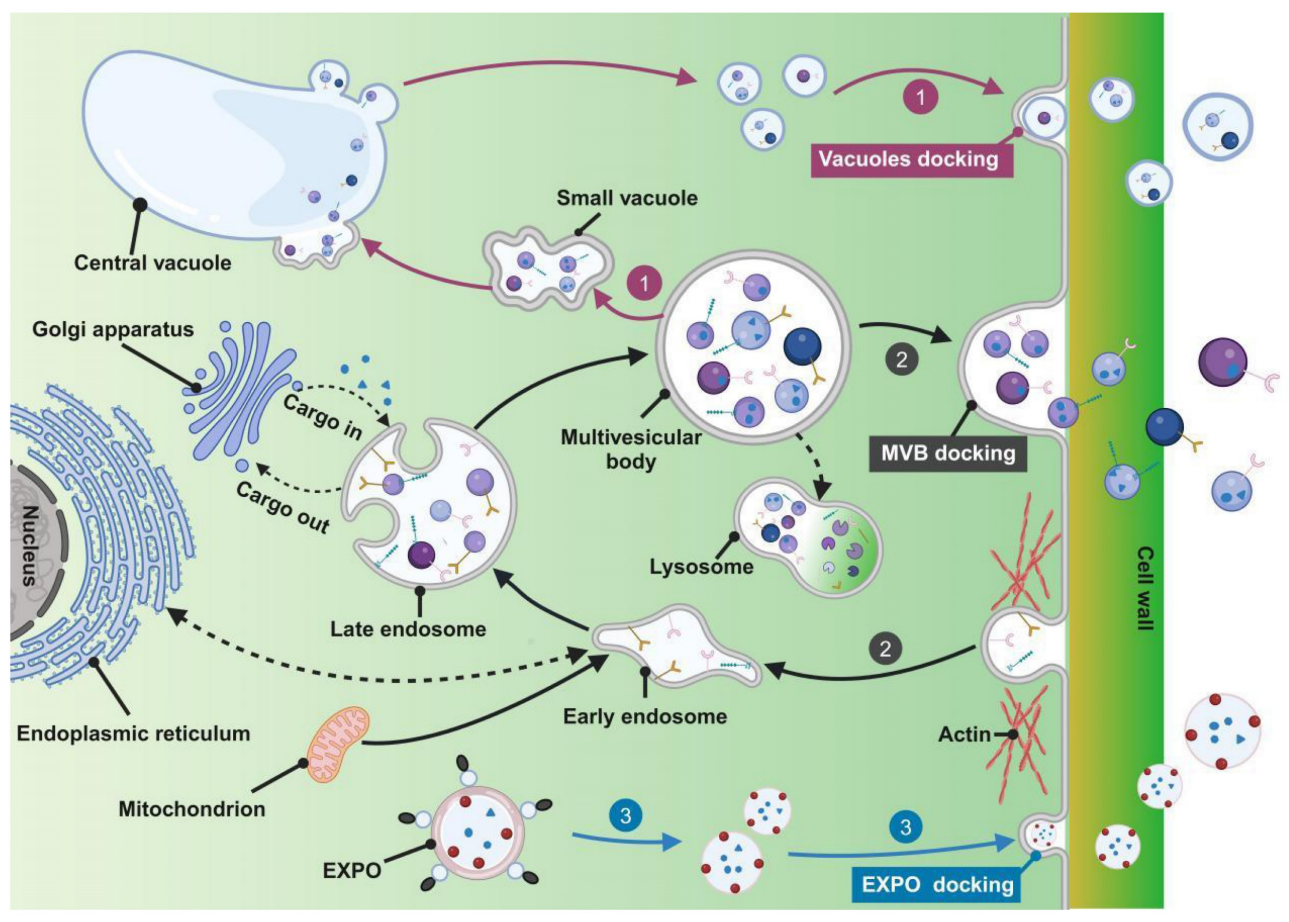
**Biogenesis of PELNs. 1)** Vacuolar pathway; **2)** MVBs pathway; **3)** EXPO pathway. (Created with Biorender.com).

The biological genesis of PELNs encompasses three pathways: the vacuolar, the multivesicular bodies (MVBs), and the exocyst-positive organelle (EXPO) pathways (**Figure 1**) 18.

The MVBs pathway is a key route for exosome formation in animal cells and is considered fundamental in PELN biogenesis. Initially, the cytoplasmic membrane undergoes inward budding, leading to endocytic vesicle formation to internalize various components including lipids, proteins, small molecules, and diverse metabolites, initiating the formation of early endosomes. Fusion of early endosomes with components from the endoplasmic reticulum, trans-Golgi network, and mitochondria facilitates cargo acquisition. Subsequent maturation of early endosomes forms late endosomes, known as MVBs, encapsulating cargo within vesicles. It is important to highlight that MVBs may fuse with lysosomes, resulting in degradation due to the presence of ubiquitinated cargo. Transport of MVBs, along with their eventual fusion with the plasma membrane and release of vesicular contents, is mediated by specific components of the microtubule scaffold, actin, and the Rab family 21-23. Alternatively, escaped MVBs may fuse with the plasma membrane to release exosomes 24.

The vacuolar pathway involves the recently discovered fusion between vacuoles containing hydrolytic enzymes and defense components with the plasma membrane. Pseudomonas syringae pv. tomato DC3000 (Pst DC3000) potentially represents a defensive signaling response to bacterial infection when expressed in plants 25. A study in Arabidopsis demonstrates that during infection by the pathogenic bacterium Pst DC3000, fusion occurs between the vacuolar membrane and the plasma membrane, releasing antimicrobial proteins and hydrolytic enzymes into the extracellular space, serving as a defense strategy against pathogenic invasion 26. Furthermore, PELNs were identified within the central vacuole of grapefruit epidermal cells 27 and plant cells contain small vacuoles (SVs) in early developmental cortical cells, harboring vesicles derived from the fusion of maturing MVBs. The central vacuole originates from the MVB-to-SV transition and subsequent fusion of SVs, suggesting potential connections between the vacuolar pathway and the MVB pathway 28.

The EXPO pathway represents an unconventional secretion route in plants characterized by a double-membrane EXPO that mediates exocytosis from the cytoplasm to the cell wall. In contrast to autophagosomes, EXPO is not induced by starvation and does not merge with endosomes or lytic compartments, instead, they fuse with the plasma membrane to release single-membrane vesicles, including exosomes, into the cell wall 29.

### 2.2 Preparations of PELNs

Unlike the methods used for isolating animal exosomes, the study of PELNs requires pretreatment of plant tissues prior to isolation and purification. In this paper, we briefly summarize the principles, advantages, and disadvantages of these techniques, as shown in **Table 1**.

#### 2.2.1 Tissue pretreatment methods

Plant materials are initially cleaned to remove dust and soil, then washed with phosphate-buffered saline (PBS) to eliminate ions and elements from tap water. Subsequently, the materials are processed to obtain the plant's juice or apoplast fluid.

Plant tissues with a high water content can be blended to extract plant juice, such as grapes and ginger 14, 30. However, while this method facilitates the separation of nanovesicles from plants, there is a potential risk of cellular damage, leading to the mixing of organelle or membrane structures with EVs 31.

Plant roots or leaves are immersed in buffer solutions such as 2-(N-morpholino) ethanesulfonic acid (MES), which closely mimic native apoplastic fluids, to obtain apoplastic fluid. A widely used technique for extracting apoplastic fluid is the infiltration-centrifugation method, which involves vacuum infiltration with an infiltration buffer, without the need for homogenization 26, 32. However, the utilization of buffer solutions may dilute the native PELN fluid, resulting in a comparatively reduced concentration of PELNs. Additionally, the addition of washing liquids to dilute the PELN fluid surrounding plant cells may distort the plant's metabolic profile 33.

#### 2.2.2 Separation and purification methods

**Figure 2:**
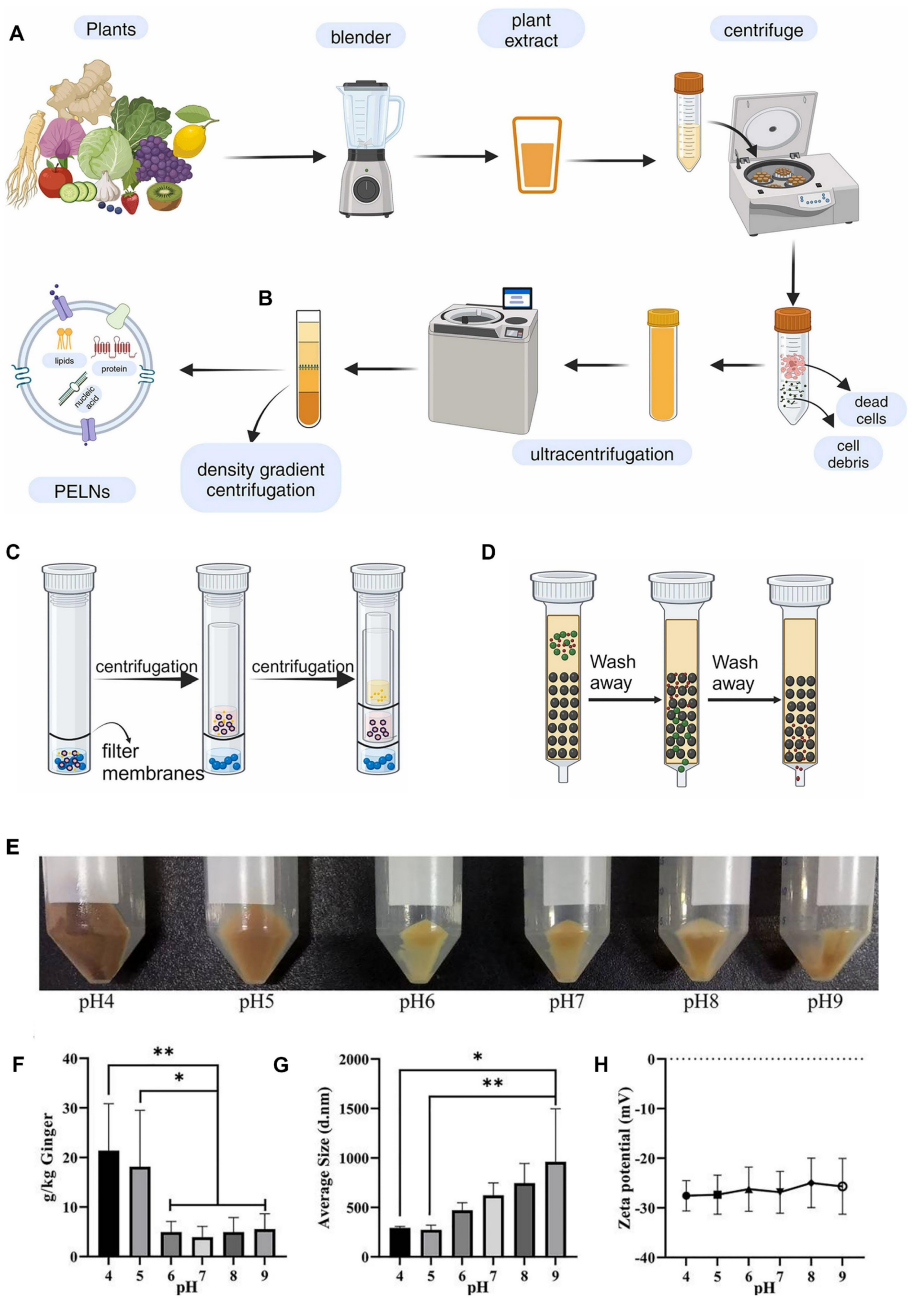
**Extraction method of PELNs and the influence of pH on PELNs extraction. (A)** Ultracentrifugation,** (B)** density gradient centrifugation, **(C)** ultrafiltration and **(D)** size exclusion chromatography method. Adapted with permission from 175, copyright 2024, Biomedicine & Pharmacotherapy. **(E)** Photomicrographs, **(F)** total yield, **(G)** average size, and **(H)** average zeta potential of ginger PELNs isolated under different pH. Adapted with permission from 43, copyright 2021, ACS Omega. PDNs: Panax PELNs.

After obtaining heterogeneous PELN mixtures through methods like blender juice extraction or tissue infiltration-centrifugation, additional steps for separation and purification are necessary. PELN separation and purification primarily employs techniques such as ultracentrifugation, density gradient centrifugation, ultrafiltration, and size exclusion chromatography (**Figure 2A-D**).

PELN extraction methods are often adapted from established exosome protocols and typically involve ultracentrifugation, effectively eliminating fibrous debris from the plant tissues 34 and facilitating PELN sedimentation 35. To enhance the purity of PELNs, the obtained precipitate is typically resuspended in PBS and subjected to a second round of high-speed centrifugation. However, this approach often leads to a decreased yield 36. While this technique lacks precision in isolating exosomes, the presence of high-molecular-weight components such as cellulose and starch in plant juice often leads to lower purity after centrifugation. Consequently, the purification of exosomes obtained after ultracentrifugation remains a frequently utilized method for PELN extraction.

Density gradient centrifugation can be considered a more precise method of ultracentrifugation, and research has also investigated its application for further purification following ultracentrifugation of PELNs 31. The primary PELN purification method is density gradient centrifugation using sucrose or iodixanol (typically at concentrations of 8%, 15%, 30%, 45%, and 60% w/v). The purified exosomes usually reside in the intermediate layer of a 30%-45% sucrose solution 10. Ginger-derived PELNs were purified from the interfaces of 8/30% and 30/45% sucrose gradients, with those at 30/45% demonstrating notable biological activity. It is noteworthy that from an initial material of 1000 g of ginger, approximately 50 mg of ginger PELNs were obtained, underscoring the necessity of purification 20. Although PELNs separated by density gradient centrifugation typically exhibit high purity, the resulting yield is often relatively low 37.

PELNs have also been extracted by ultrafiltration, followed by size-exclusion chromatography purification and pre-concentration of elution fluids, with the separation process monitored using capillary electrophoresis 38. A study compared the efficiency of PELN separation using ultrafiltration and ultracentrifugation, and found that the ultrafiltration method retained the optimal PELN morphology, albeit with the highest level of protein contamination 39.

Size exclusion chromatography is a separation method based on particle fluid dynamics, primarily used to separate vesicles of varying sizes from other biomolecules using gel filtration matrices or resin filtration 40. The orange-derived products could be subdivided into either smaller (<50 nm) or larger (>150 nm) PELNs through size exclusion chromatography 41. This method yields PELNs with high purity, characterized by minimal impurities from non-nanoparticle proteins and other large molecules, thereby preserving their structural integrity. However, it has a low yield, requires specialized equipment, and involves relatively complex procedures 42.

The extracted PELNs can be significantly influenced by the adjusted pH of the plant juice before extraction. For instance, compared to ginger juice with a pH of 5, adjusting ginger juice to a pH of 4 yields more PELNs with increased biological activity content and miRNA (**Figure 2E-H**) 43. While numerous methodologies have been developed for the extraction and isolation of exosomes, achieving consistent levels of purity and concentration remains a challenge. Even with the same isolation method, the yields of PELNs can vary significantly. For instance, ginseng PELNs extracted using ultracentrifugation and density gradient centrifugation have shown different yields across studies (168 mg protein/kg ginseng and 500 mg protein/kg ginseng) 44, 45. These differences may arise from factors such as the plant source, harvest season, and freshness. Additionally, studies use diverse units to quantify PELN yield (**Table 1**). Therefore, standardizing the units of measurement for PELN yield is recommended to facilitate easier comparison of yields obtained from different isolation methods.

### 2.3 Composition of PELNs

Under physiological conditions, exosomes conventionally transfer functional biomolecules like mRNA, microRNA, long non-coding RNA, circular RNA, and DNA between cells, thus facilitating intercellular communication 46. Due to the shared evolutionary origin of plants and animals, there are similar components in PELNs and mammalian EVs 43. However, the types and quantities of cargo in PELNs are fewer compared to those in mammalian EVs, indicating higher safety and easier functional resolution of PELNs. For instance, PELNs extracted from grapes contain 28 proteins and 96 miRNAs in contrast to the typical profile of mammalian exosomes, which commonly feature over 1000 proteins and 100-300 miRNAs 47. Furthermore, several studies have shown that components within PELNs can target human genes and offer therapeutic effects (**Table 2**).

#### 2.3.1 Proteins

Penetration 1 (PEN1), PEN3, and Tetraspanin-8 (TET-8) are potential protein markers for identification in PELNs 48. PEN1 co-localizes with the amphiphilic styryl dye FM4-64 outside the plasma membrane, accumulating extracellularly in papillae, suggesting its secretion via exosomes 49. TET-8 shares homology with mammalian exosomal markers (CD63, CD81, and CD9), and co-localizes with intracellular MVB markers in plant cells, suggesting its potential utility as a marker for plant exosome-like vesicles 50.

Moreover, PELNs generally exhibit a lower protein concentration, predominantly consisting of cytoplasmic and transmembrane proteins 18. Analysis of ginger-derived PELNs revealed the presence of proteins like actin, proteases, and various membrane proteins, including transport proteins such as aquaporins and chloride ion channels, which may be more relevant to the endocytosis function of PELNs 20. However, substantive studies on the proteins in PELNs are limited, with most relying on proteomics and bioinformatics to predict the functions of the identified proteins. For instance, Wang et al. conducted protein structure analysis and GO/KEGG enrichment analysis on proteins extracted from bitter melon PELNs, suggesting that the proteins may possess antioxidative capabilities, potentially alleviating disease symptoms through their antioxidative effects 51.

#### 2.3.2 Lipids

Lipids, a crucial yet relatively underexplored component of exosomes, encompass sphingolipids, phosphatidic acid (PA), sphingomyelin (SM), phosphatidylserine (PS), and cholesterol 22. Lipids not only contribute to the structural integrity of PELN membranes but also actively participate in PELN formation and release 52. Lipidomic analysis of PELNs has revealed two distinct classes of lipids, named phospholipids and glycerol lipids, which are involved in the uptake and various biological functions of PELNs. However, the presence of cholesterol in PELNs has not been confirmed 10. Interestingly, the lipid content of PELNs may be different from that of their parent plants. For example, PELNs from grapefruits exhibited a lipid composition similar to grapefruit tissues, with a higher proportion of sphingolipids, particularly hexosylceramides and ceramides 53.

PELN-derived lipids are believed to possess biotherapeutic effects. For example, ginger PELNs contain abundant PA, which induces Foxa2 expression in intestinal epithelial cells, thereby preventing high-fat diet-induced obesity and insulin resistance 54. Additionally, PA directly interacts with the HBP35 protein in Porphyromonas gingivalis conferring antibacterial properties 55.

Differential centrifugation and ultrasonication were used to extract lipids from PELNs, which can be reassembled into lipid-based PELNs as a potential drug delivery platform. These lipid PELNs demonstrate minimal toxicity, possess the potential for targeted delivery through modifications, and can deliver various agents. For instance, grapefruit lipid PELNs loaded with a Stat3 inhibitor demonstrate remarkable in vitro uptake capacity and targeted delivery to brain tumors, effectively inhibiting tumor growth 56.

#### 2.3.3 Nucleic acids

Nucleic acids encapsulated in EVs act as mediators for intracellular communication and constituents of diverse signaling pathways. Among these, miRNAs have been shown to have a central role in the therapeutic effects of most EV treatments 57. Notably, discrepancies in the types of nucleic acids present may exist between PELNs and their source plant tissues. For instance, ginger-derived PELNs have a more abundant miRNA cargo compared to ginger tissues, coupled with a reduced tRNA content 58. Bioinformatics enables the prediction of miRNA targets within the human genome, facilitating the investigation of plant-derived miRNAs in human diseases. For example, Osa-miR-530-5p in ginger PELNs indirectly hinders ORF1b synthesis by targeting the ribosomal slippage site in the ORF1ab gene, thereby impeding the replication of severe acute respiratory syndrome coronavirus 2 (SARS-CoV-2) 59.

The analysis of 11 different PELNs, including coconut, ginger, and hamimelon, identified 418 distinct miRNAs. The abundant miRNAs present in multiple plants are referred to as frequent miRNAs, whereas those existing in individual plant species are known as rare miRNAs. Notably, "frequent" miRNA categories are less numerous than "rare" miRNA categories, despite the former exhibiting higher cumulative expression levels 60. Certain miRNAs present in substantial quantities across multiple plant species potentially serve as plant-specific miRNAs.

#### 2.3.4 Secondary metabolites

PELNs transport small secondary metabolites produced by plants, such as flavonoids, saponins, polysaccharides, alkaloids, etc. 61. Active metabolites, specifically 6-gingerol, and 6-shogaol, have been identified in ginger PELNs 20 and PELNs isolated from sucrose gradients of 8%/30% exhibited higher concentrations of shogaol compared to those between 30%/45%, suggesting potential specific variations based on PELN density 62.

Secondary metabolites in PELNs may also be used in the treatment of human diseases. Our previous research has extracted PELNs from the embryonic layer of Flos Sophorae Immaturus and identified rutin, a crucial therapeutic component for the treatment of spinal cord injuries 63. The metabolites encapsulated in PELNs demonstrate a propensity to enhance cellular absorption and accumulation within recipient cells. For instance, the β-glucans in oat-derived PELNs facilitate a dose-dependent uptake of PELNs by microglia 64.

### 2.4 Uptake mechanism of PELNs

PELNs can be internalized by diverse mammalian cells including cancer cells (breast, lung, colon, and glioma cells), lymphocytes (T cells and B cells), neural cells (neurons, microglia, astrocytes, and brain microvascular endothelial cells), as well as normal human skin keratinocytes (HaCat cells) 56, 60, 65. Based on current reports, PELNs demonstrate uptake rates exceeding 40% in most cell lines 66-69. Even in difficult-to-transfect T cells and B cells, they achieve uptake rates of 14.1% and 19.8%, respectively 56.

**Figure 3:**
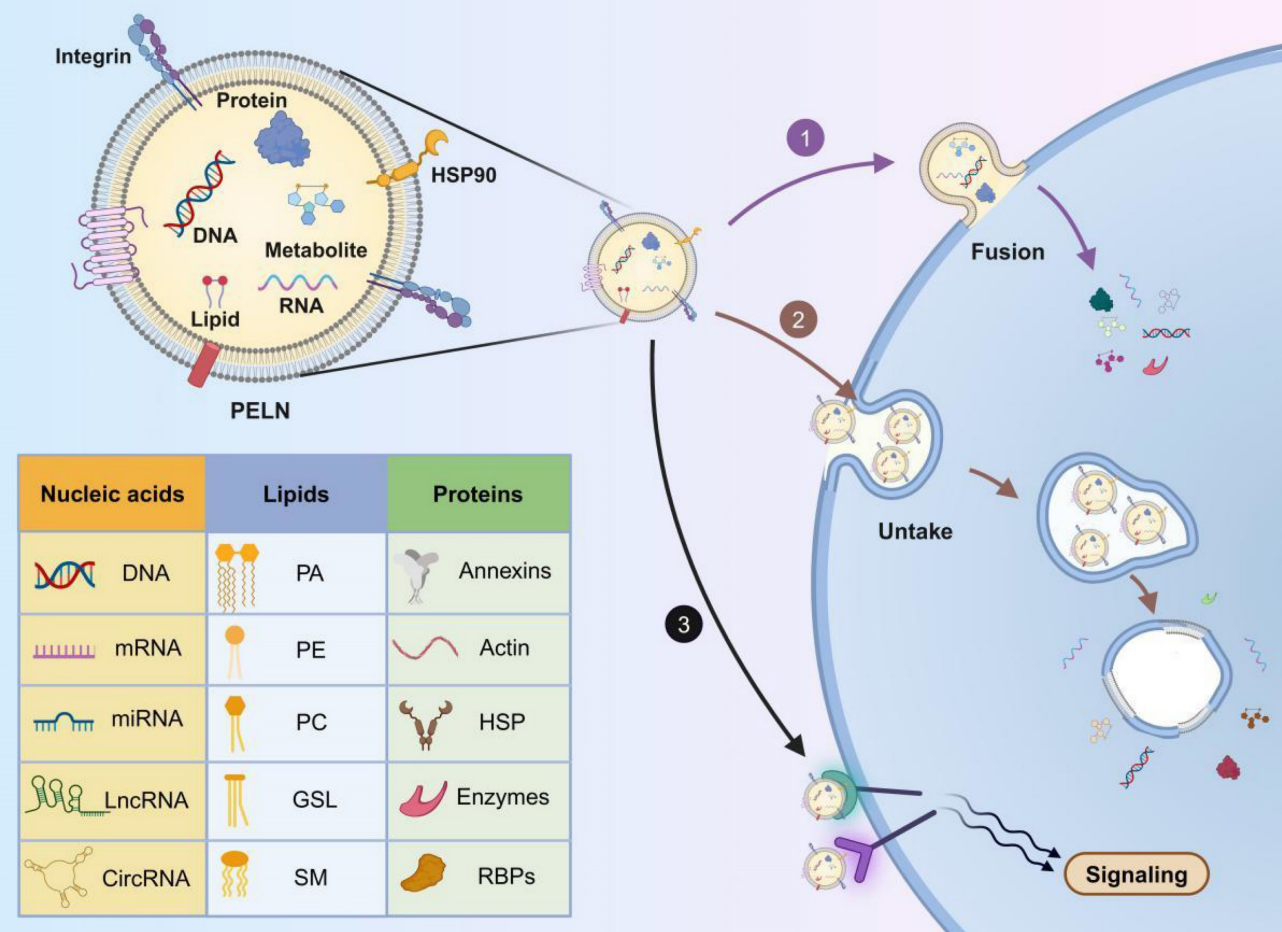
**The composition and uptake of PELNs. 1)** Fusion with the target cell membrane; **2)** internalization through endocytosis into target cells; **3)** binding to receptors on the target cell membrane. PA: phosphatidic acid; PC: phosphatidylcholine; PE: phosphatidylethanolamine; GSL: glycosphingolipids; SM: sphingomyelin; HSP: heat shock proteins; RBPs: RNA binding proteins. Created with Biorender.com.

It is widely accepted that PELNs can enter target cells through three distinct mechanisms: ① direct fusion with the target cell membrane, releasing their cargo into the cytoplasm. This fusion involves the formation of a hemi-fusion stalk between the hydrophobic lipid bilayers of the PELN and the plasma membrane, followed by expansion into a cohesive structure and is likely facilitated by the SNARE and Rab protein families 70; ② internalization through endocytosis into target cells, followed by the release of cargo into the cytoplasm. This uptake process is rapid and temperature-sensitive, with lower temperatures attenuating internalization 71. Common endocytic pathways, such as clathrin-mediated endocytosis, lipid raft-associated membrane invagination, and caveolin-dependent endocytosis, mediate exosome internalization 72; ③ binding to receptors on the target cell membrane, initiating receptor-ligand interactions and downstream signaling cascades to activate the target cell 73 (**Figure 3**).

Transport proteins located on the surface of PELNs may influence their uptake mechanisms. Studies have indicated that the endocytosis of garlic-derived PELNs by HepG2 cells depends on the interaction between the surface protein II agglutinin on PELNs and CD98 on HepG2 cells 74. Therefore, the role of transport proteins on the surface of PELNs in cellular uptake mechanisms warrants further exploration. Additionally, PELN uptake may depend on time and dosage. Ginseng-derived PELNs are internalized by bone marrow mesenchymal stem cells (BMSCs) in a time-dependent manner, reaching a peak after 12 hours 75, whereas PELNs from blueberry demonstrate dosage-dependent internalization into the human stable endothelial cell line EA.hy926 76.

Understanding the intracellular transport pathway and fate of PELNs following cellular uptake is crucial for ongoing research. Currently, only one study has observed that grapefruit-derived PELNs co-localize with endosomes and lysosomes within HaCaT cells six hours post-uptake 77. This suggests a potential pathway where PELNs might be degraded by lysosomes within the cell. Overall, research on the mechanisms underlying the intracellular fate of PELNs remains incomplete. Elucidating the molecular mechanisms involved in their internalization process will pave the way for more effective treatments and drug delivery strategies.

### 2.5 Distribution of PELNs

The distribution of PELNs within living organisms is influenced by the method of administration including oral ingestion, intravenous injection, transdermal delivery, nasal administration, intraperitoneal injection, and intramuscular injection. Among these, upon oral administration, PELNs are primarily distributed in the gastrointestinal tract. For example, after mice were given orange PELNs by gastric gavage, fluorescence was detected in the gastrointestinal tract after 6 hours, and significant metabolism was observed within 24 hours 41, 78. Interestingly, ginger-derived PELNs are also distributed within the gastrointestinal tract and are taken up by intestinal bacteria, affecting both the microbiota composition and host physiology 79. Therefore, oral administration of PELNs is well-suited for gastrointestinal conditions such as gastrointestinal cancer and colitis. PELNs exhibit good stability in gastric and intestinal environments. In simulated gastric acid (pH=2.0, supplemented with pepsin), yam-derived PELN vesicles maintained their quantity despite an increase in particle size, retaining their functional targeting and communication roles 80. Moreover, Zhang et al. investigated the variations in size and surface charge of grapefruit, carrot, and ginger PELNs in simulated gastric acid and intestinal fluid (pH=6.5, supplemented with bile and pancreatin). They observed some subsets separated or merged for PELNs, with a decrease in negative charge in simulated intestinal fluid, thereby enhancing their resistance to gastrointestinal digestion 81. In summary, PELNs demonstrate robust resistance to degradation in gastric and intestinal fluids, underscoring oral administration as a crucial delivery method for PELNs.

Intravenous injection avoids the first-pass effect and allows for the distribution and targeting of PELNs in tissues outside the gastrointestinal tract. PELNs derived from Panax notoginseng targeted the brain in rats with cerebral ischemia 8 to 12 hours post-intravenous injection 31. Similarly, bitter melon-derived PELNs exhibited targeted accumulation in tumors after intravenous administration in tumor-bearing mice, with sustained high levels even after 72 hours, suggesting a potentially extended in vivo half-life 82. Most bitter melon PELNs accumulate in the liver, indicating predominant metabolism through the liver 82. However, not all PELNs can effectively target organ lesions, necessitating engineering modifications to enhance their targeting ability after intravenous injection. For instance, modifying the outer membrane of ginseng-derived PELNs improved their targeting of inflammatory areas 83. The blood circulation characteristics of PELNs after intravenous injection vary depending on their source. For instance, ginger lipid-derived PELNs remain stable and detectable in the bloodstream for up to 48 hours post-injection 66. Conversely, corn-derived PELNs are rapidly cleared from the systemic circulation following intravenous administration. However, when corn PELNs are modified with polyethylene glycol (PEG), they exhibit significantly enhanced distribution in tumor tissues and prolonged circulation time 84. Therefore, it is important to investigate the blood circulation characteristics of PELNs, and explore modifications to extend their systemic circulation time in PELN research.

Various administration routes have been investigated for PELNs, including transdermal, nasal, intraperitoneal, and intramuscular routes. For instance, PELNs derived from broccoli have demonstrated superior penetration through both the stratum corneum and dermis of pig skin when administered transdermally 15. Grapefruit-derived PELNs, upon nasal administration, were found to distribute exclusively in the brain 56, 85 and lungs 56. Furthermore, following intraperitoneal injection, grapefruit PELNs predominantly distributed in liver, lung, kidney, and spleen tissues, similar to intravenous injection 56. Conversely, intramuscular injection of grapefruit PELNs resulted in their distribution exclusively within muscle tissue. Therefore, specific administration methods can be selected based on the site of disease manifestation 56.

The in vivo biodistribution mechanisms of PELNs sourced derived from different plant sources are complex, necessitating further foundational research exploration.

## 3. Biotherapeutic applications of PELNs

PELNs present substantial potential in treating human diseases owing to their anti-inflammatory 86, antiviral 87, antioxidant 88, anti-tumor 89, gut microbiota regulation 90, insulin resistance regulation 91, anti-osteoporotic 92 and regenerative effects 93. Compared to traditional cell therapy or drug therapy, PELNs offer significant advantages including lower immunogenicity, improved targeting, and scalability. Furthermore, the modification or engineering of PELNs may enhance their therapeutic capabilities for specific diseases.

PELNs derived from fruits, vegetables, and herbs collectively exhibit common biological therapeutic effects, such as anti-inflammatory properties, anti-tumor activity, and regulation of gut microbiota (**Table 3**). Additionally, PELNs from these sources each display distinct biological activities. Fruit-derived PELNs exhibit antioxidant effects, vegetable-derived PELNs show antiviral properties and regulation of insulin resistance, whereas herb-derived PELNs possess anti-osteoporosis and regenerative properties (**Table 4**).

### 3.1 Anti-inflammatory activity of PELNs

PELNs can modulate macrophages, key participants in the pathogenesis of numerous chronic inflammatory and autoimmune diseases. For example, pueraria lobata PELNs promote the polarization of M1 macrophages towards the M2 phenotype by downregulating the expression of pro-inflammatory genes 94. Additionally, miR-396e in garlic-derived PELNs regulated the expression of PFKFB3, influencing metabolic reprogramming in macrophages and alleviating inflammation in adipocytes of obese mice through crosstalk between macrophages and adipocytes 95.

Intriguingly, some studies suggest that oral administration of PELNs exhibits notable therapeutic efficacy against intestinal inflammation. Oral administration of grape and orange PELNs promotes mucosal epithelial regeneration and rapid recovery of the entire intestinal structure in dextran sulfate sodium (DSS)-induced colitis mice 14, 41, 96. Similar effects have been observed in PELNs derived from celery 97, allium tuberosum 98, potato 99, and bitter melon 51. The findings underscore the pivotal role of PELNs in promoting intestinal function, thereby supporting the importance of increased fruit and vegetable consumption and adherence to a well-balanced diet, such as the Mediterranean diet.

Furthermore, garlic-derived PELNs containing PA (36:4) interact with BASP1 in microglial cells, inhibiting c-Myc-mediated STING expression and reducing the expression of inflammatory cytokines, thereby reducing inflammation induced by a high-fat diet 100. Oat bran-derived PELNs ameliorate alcohol-induced brain inflammation by modulating the assembly of the HPCA/Rab11a/dectin-1 complex 64. These findings confirm that dietary-derived factors have the potential to impact brain health through their anti-inflammatory effects.

### 3.2 Anti-tumor activity of PELNs

Various PELNs have demonstrated potent anti-tumor effects including promoting apoptosis, cell cycle arrest, inhibition of cell proliferation, inducing mitochondrial damage, and inhibiting epithelial-mesenchymal transition. For example, lemon PELNs induce ROS generation, upregulate GADD45α expression leading to cell cycle arrest in the S phase, and induce apoptosis in gastric cancer cells 78. Furthermore, PELNs derived from corn have been shown to stimulate the release of inflammatory factors from Raw264.7 cells, leading to the inhibition of colon26 cell proliferation and suppression of subcutaneous colon26 tumor growth in mice 101.

**Figure 4:**
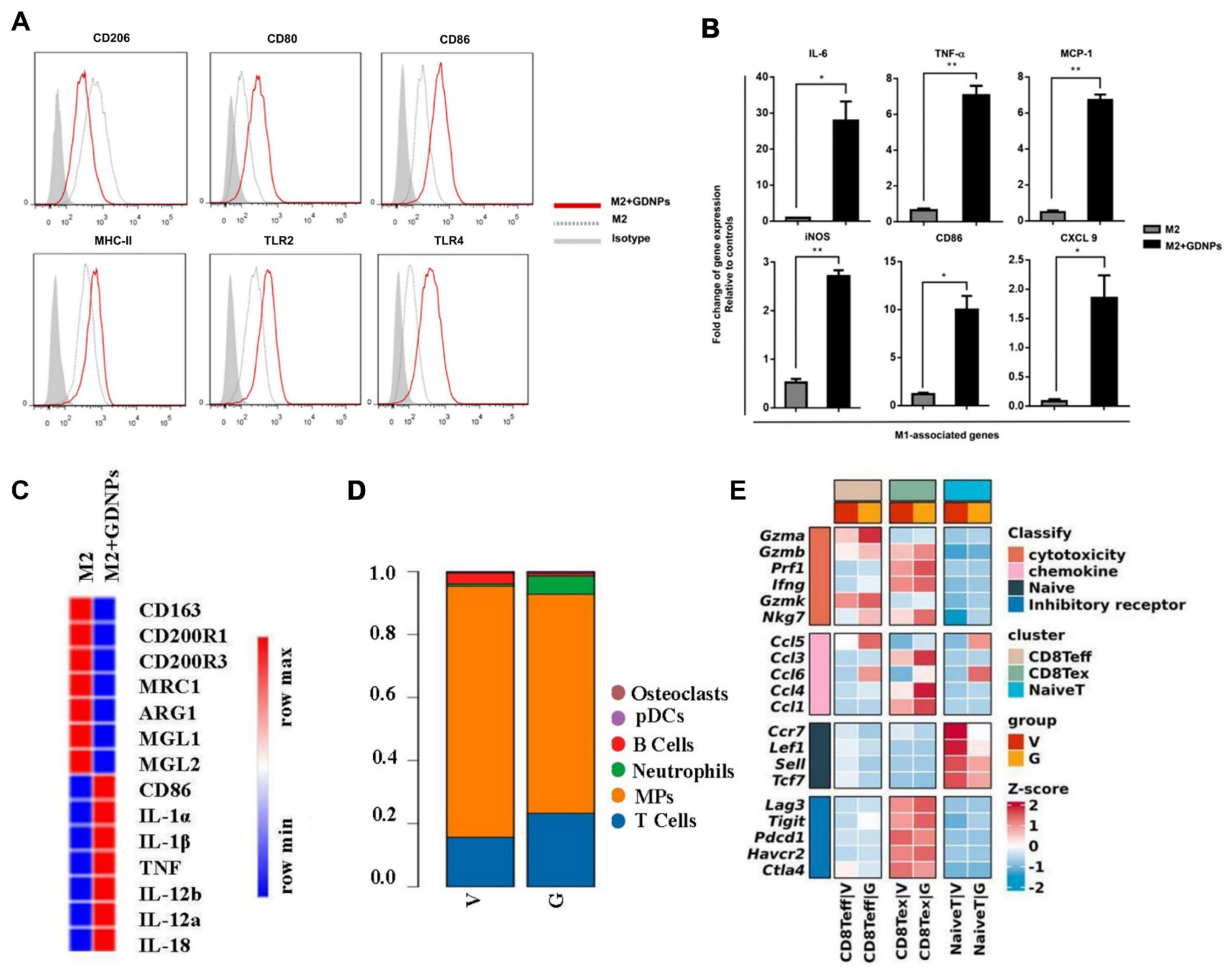
**Impact of ginseng-derived PELNs on TAMs and modulation of immune cells in TME. (A)** Flow cytometry analysis of the effect of ginseng-derived PELN treatment on the surface marker expression of M2 macrophages. **(B)** Quantitative mRNA expression of M1 marker genes and M2 marker genes. Adapted with permission from 44, copyright 2019, Journal for ImmunoTherapy of Cancer. **(C)** Heat map analysis of the effect of ginseng-derived PELNs on M1-M2 related gene expression in M2-like macrophages. **(D)** Single-cell sequencing analysis of the effect of G (ginseng-derived PELNs) and V (Vehicle) on the proportion of immune cells in MC38 mouse cancer.** (E)** Heat map showing the expression of cytotoxicity, chemokine, naive, and inhibitory receptor in CD8+ T cell subsets (naive T, CD8 Teff, and CD8 Tex) in G and V groups. Adapted with permission from 103, copyright 2023, Journal of Experimental & Clinical Cancer Research.

Several notable studies highlight the therapeutic impact of ginseng-derived PELNs on both "cold" and "hot" tumors by modulating tumor-associated macrophages (TAMs) 44, 45, 102, 103. Ginseng-derived PELNs induce the polarization of TAMs from M2 to M1 phenotype through a TLR4/MyD88-dependent mechanism, resulting in elevated total ROS production and increased apoptosis in B16F10 melanoma cells in mice, thereby decelerating tumor progression (**Figure 4A-C**) 44. Additionally, ginseng-derived PELNs reprogram TAMs and enhance the function of CD8 Teff via the mTOR-T-bet axis to regulate arginase-1 (ARG1) release thereby downregulating immune checkpoint expression on T cells in the tumor microenvironment (TME) and ameliorating T cell exhaustion (**Figure 4D-E**). This recruitment of CD8+ T cells inhibits the growth of hot tumors, with a marked synergistic effect when combined with PD-1 antibody therapy. Notably, combining ginseng-derived PELNs with PD-1mAb optimizes the TME by increasing tumor-infiltrating lymphocytes, thus converting cold tumors into hot tumors 102, 103. Therefore, PELNs, epitomized by ginseng, offer a strategy for targeting TAMs in cancer therapy.

### 3.3 Gut microbiota regulation of PELNs

Certain PELNs can regulate gut microbiota abundance, thereby maintaining gut homeostasis 67, 69, 90, 104-106. For example, the p-miR2916-p3 within garlic-derived PELNs specifically promotes the growth of Bacteroides thetaiotaomicron, consequently suppressing DSS-induced colitis 90. Rgl-miR-7972 within Rehmanniae radix PELNs have been reported toinhibits Escherichia coli biofilm formation by targeting the virulence gene sxt2, thereby alleviating LPS-induced lung inflammation and recovering gut microbiota dysbiosis 105. Therefore, the miRNAs present in PELNs could potentially play a crucial role in regulating the composition of gut microbiota.

An intriguing study revealed the capacity of garlic-derived PELNs to train human gut Akkermansia muciniphila (A. muciniphila) to release outer membrane vesicles (OMVs), leading to elevated expression of insulin receptor substrates (IRS1 and IRS2) in microglial cells. This offers a promising avenue for addressing high-fat diet-induced type 2 diabetes and alleviating brain inflammation 107. In addition, portulaca oleracea L. PELNs demonstrate a dose-dependent modulation of gut microbiota abundance, thus ameliorating intestinal and pulmonary inflammation at the gut-lung axis level 106. The intestinal microbiota plays a critical role in establishing connections between the intestine and other organs, including the brain-gut axis, liver-gut axis, and lung-gut axis, thus the regulatory influence of PELNs on the intestinal microbiota may also extend to other organs associated with the intestine.

**Figure 5:**
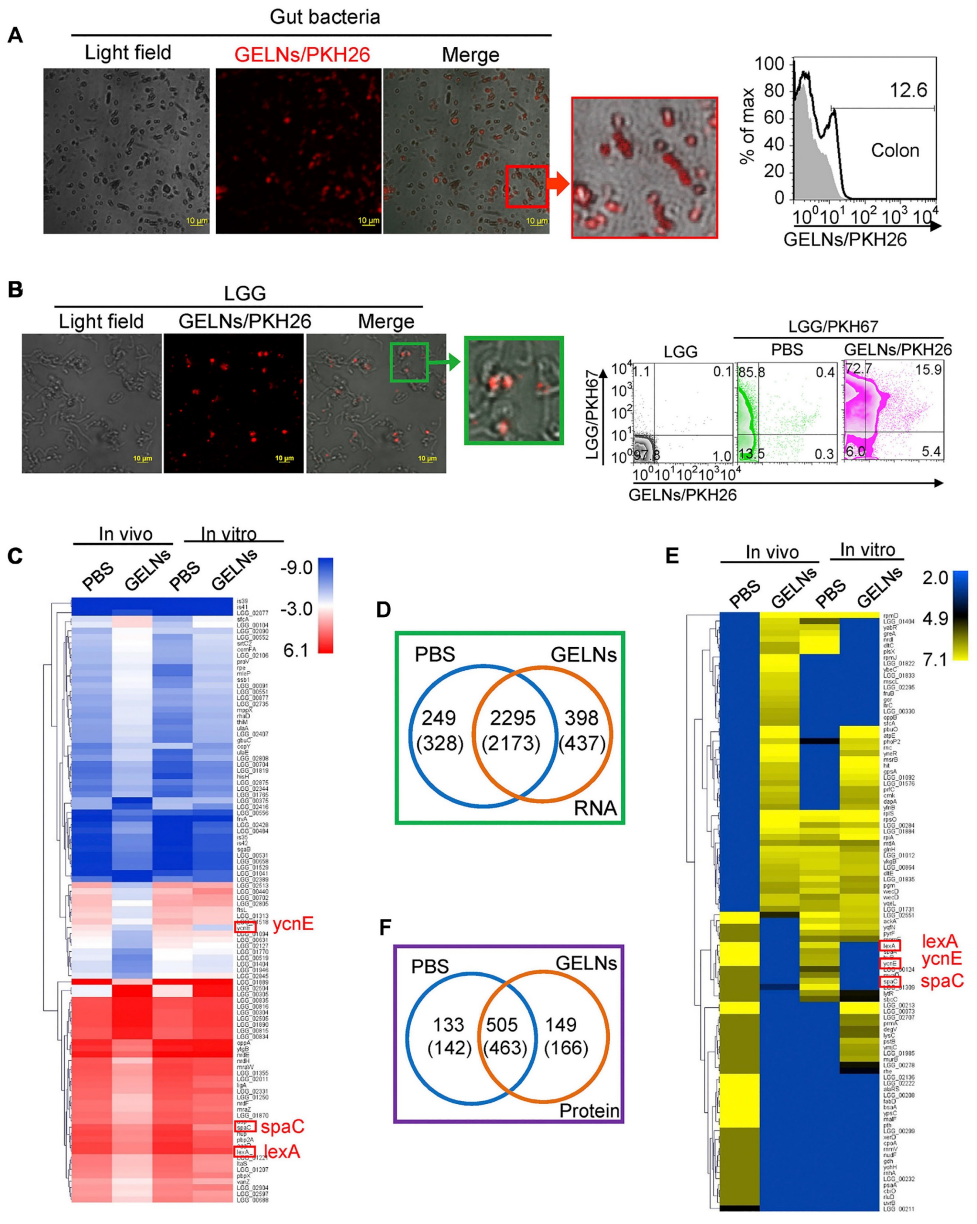
** Absorption of ginger PELNs by LGG and regulation of LGG mRNA and proteins. (A)** Representative confocal microscopy images (scale bar: 10 μm) and flow cytometry quantification of fecal samples from mice fed with ginger PELNs; **(B)** Confocal microscopy images (scale bar: 10 μm) and flow cytometry quantification of the colocalization between ginger PELNs and LGG colonies; **(C)** Heatmap illustrating the influence of GELNs on LGG mRNA expression as determined by next-generation sequencing; **(D)** Venn diagram of all mRNA detected in LGG. Numbers in parentheses represent in vitro results. **(E)** Heatmap of the impact of GELNs on LGG protein expression based on LC-MS data; **(F)** Venn diagram of all proteins detected in LGG. Adapted with permission from 79, copyright 2018, cell host & microbe.

Notably, ginger PELNs demonstrate promising capabilities in mitigating intestinal inflammation by selectively regulating the mRNA and proteins within the gut microbiota (**Figure 5**). It has been reported that mdo-miR7267-3p in ginger PELNs efficiently target the monooxygenase ycnE of LGG in a lipid-dependent manner. This targeted interaction subsequently activates antimicrobial immunity and promotes tissue repair at the mucosal surface, thereby improving intestinal barrier function in a DSS-induced colitis mouse model 79.

Additionally, PELNs indirectly impact disease progression by modulating the metabolites of gut microbiota. For example, pueraria lobata PELNs accelerate the degradation of trimethylamine-N-oxide (TMAO), a gut microbiota metabolite, leading to increased cellular autophagy, ultimately promoting the osteogenic differentiation and functionality of human bone marrow mesenchymal stem cells (hBMSCs) 92. The metabolites produced by the gut microbiota have garnered considerable attention in recent research and the convergence of these metabolites with PELNs presents an exciting prospect for further exploration.

### 3.4 Antioxidant activity of fruit-derived PELNs

A variety of antioxidant compounds are found in fruit-derived PELNs, such as ascorbic acid, catalase, anthocyanin, and glutathione, which may account for their unique antioxidant activity 53, 108, 109. Studies have reported that PELNs extracted from a juice mixture of asparagus, grape, kiwi, cherry, blood orange, orange, tomato, papaya, grapefruit, lemon, mango, and bergamot significantly reduced the levels of ROS and malondialdehyde (MDA) in mice subjected to H_2_O_2_-induced oxidative stress 110. Oral administration of pomegranate PELNs reduced hepatic oxidative stress, preventing intestinal leakage, thus suggesting their potential as antioxidants for liver or intestinal protection 111. Notably, the miRNAs present in blueberry PELNs also possess anti-inflammatory and antioxidant properties 76, implying that the antioxidative effects might not solely derive from secondary metabolites. This underscores the need for further investigation into the constituents of PELNs.

### 3.5 Antiviral activity of vegetable-derived PELNs

**Figure 6:**
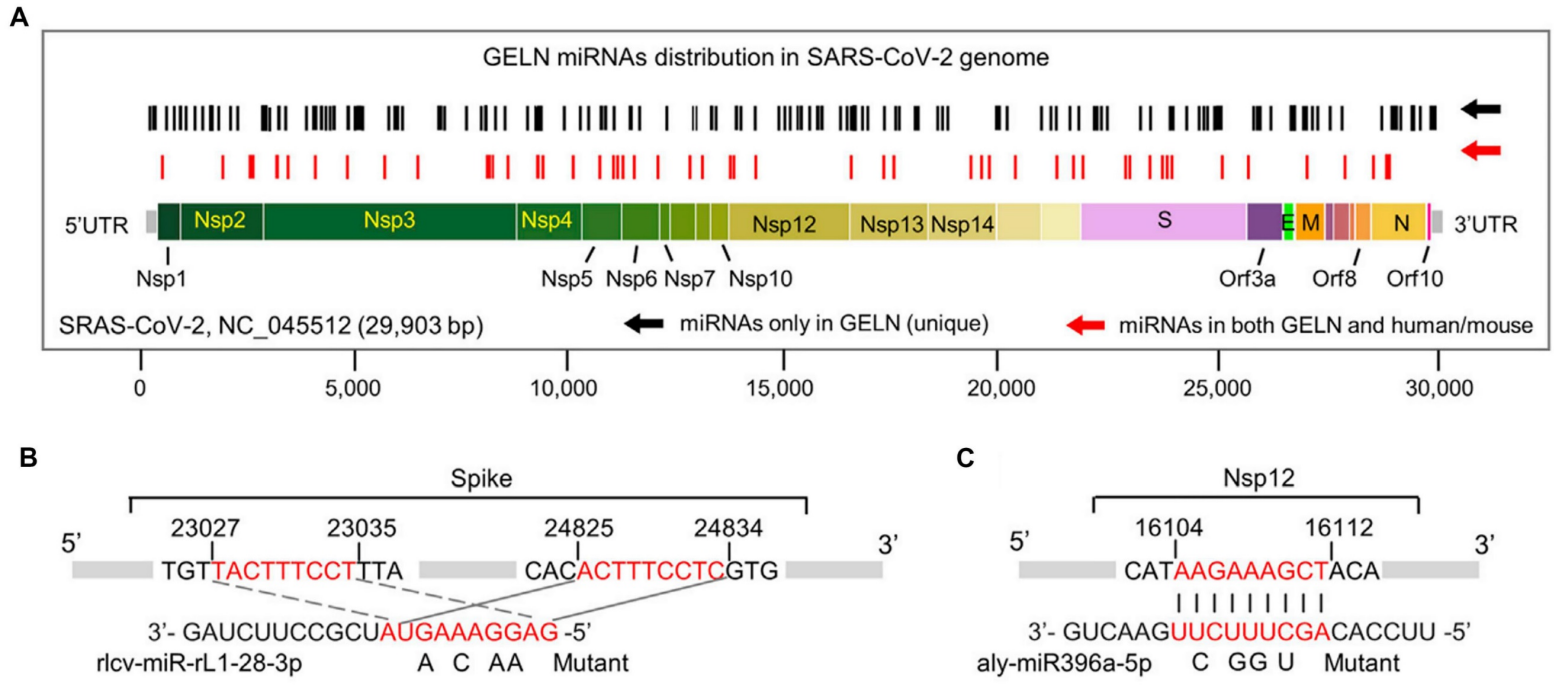
**The hypothetical targeting of SARS-CoV-2 RNA by ginger PELN miRNA. (A)** Schematic representation and positioning of putative binding sites for ginger PELN miRNAs across the SARS-CoV-2 genome. **(B-C)** Predicted complementary pairing between target regions within the spike gene (B) and Nsp12 gene (C), and ginger PELN rlcv-miR-rL1-28-3p (B) and aly-miR396a-5p (C), respectively. The miRNA seed matches within the target RNAs are mutated as indicated. GELN: ginger PELN; UTR: untranslated region. Adapted with permission from 58, copyright 2021, Molecular Therapy.

The treatment of COVID-19 induced by SARS-CoV-2 remains particularly challenging, especially in patients with comorbidities 112. Ginger, recognized for its dual role in medicine and culinary applications, has demonstrated significant potential through the miRNAs contained within ginger-derived PELNs to target RNA from the SARS-CoV-2 virus (**Figure 6**). For instance, osa-miR-530-5p in ginger PELNs indirectly inhibits the synthesis of ORF1b by targeting the ribosomal slippage site in the ORF1ab gene, thereby preventing SARS-CoV-2 replication 59. Moreover, rlcv-miR-rL1-28-3p and aly-miR396a-5p in ginger PELNs mediate the inhibition of spike genes and Nsp3 expression, thereby suppressing the cytopathic effects induced by exosomes secreted from lung epithelial cells infected with COVID-19 58. Ginger, recognized for its dual role in medicine and culinary applications, has demonstrated significant potential through the miRNAs contained within ginger-derived PELNs to target RNA from the SARS-CoV-2 virus.

### 3.6 Regulation of insulin resistance by vegetable-derived PELNs

Ginger PELNs demonstrate regulatory effects on insulin resistance and obesity. They can increase the expression of miR-375 and VAMP7 in high-fat diet mice small intestinal tissue, and inhibit the expression of the aromatic hydrocarbon receptor, thereby improving host glucose tolerance and insulin response 91. Furthermore, PA in ginger-derived PELNs induces Foxa2 expression in intestinal epithelial cells, thereby preventing high-fat diet-induced obesity and insulin resistance 54. Thus, improved insulin resistance by vegetable-derived PELNs is mainly achieved through the regulation of intestinal function, thereby indirectly impacting metabolic processes.

### 3.7 Regenerative effect of herb-derived PELNs

Herbs have long been used for disease treatment since ancient times but herb-derived PELNs, as novel nano derivatives, may possess different compositions and mechanisms compared to the herbs, necessitating further research. Herb-derived PELNs demonstrate regenerative properties by promoting skin healing, stimulating nerve regeneration, and enhancing angiogenesis. Ginseng and wheat PELNs can promote wound healing by enhancing the migration of endothelial cells and facilitating angiogenesis 93, 113. Dandelion-derived PELNs can neutralize exotoxins produced Staphylococcus aureus. When incorporated into gelatin methacryloyl hydrogels, these PELNs are continuously released, facilitating the healing process in wounds infected with Staphylococcus aureus exotoxins 114. These findings suggest potential applications in the treatment of skin wounds. In addition, ginseng-derived PELNs promote neurodifferentiation while enhancing neural regeneration through the promotion of neurotrophic factors and influencing the Ras/Erk pathway 75.

### 3.8 Anti-osteoporotic effect of herb-derived PELNs

Herb-derived PELNs have anti-osteoporotic properties, primarily by regulating the differentiation of osteoblasts and osteoclasts. Reportedly, Pueraria lobata PELNs can promote osteogenic differentiation of hBMSCs by enhancing intracellular autophagy, significantly alleviating osteoporosis in rats 92. Ginseng-derived PELNs significantly suppress IκBα, c-JUN N-terminal kinase, and ERK signaling pathways in receptor activator of nuclear factor-kappa B ligand (RANKL)-induced osteoclasts, thereby regulating genes associated with osteoclast maturation. Consequently, ginseng-derived PELNs exhibit inhibitory effects on osteoclast differentiation in an LPS-induced bone resorption mouse model 115.

In summary, herb-derived PELNs possess unique therapeutic functions that distinguish them from PELNs obtained from other sources, potentially attributed to the inherent therapeutic properties of the herbs.

## 4. Drug delivery applications of PELNs

Synthetic nanoparticles, such as liposomes, are currently the preferred carriers for drug delivery. Recent research indicates that PELNs, similar to synthetic nanoparticles, possess innate capabilities for drug delivery 19. PELNs and liposomes share fundamental characteristics, including a lipid bilayer structure enabling delivery of both hydrophilic and hydrophobic cargoes 37. However, PELNs offer several key advantages over liposomes: ① Synthetic liposomes may induce adverse effects like cellular stress 116, 117, whereas PELNs demonstrate greater biocompatibility and reduced toxicity. For example, Wang et al. showed that intravenous administration of grapefruit PELNs and DOPE liposomes at equivalent doses resulted in liver function damage only with liposomes 56. ② Unlike synthetic nanoparticles used solely as drug carriers, PELNs possess inherent therapeutic properties due to their natural cargo. ③ PELNs exhibit enhanced cellular uptake; a recent study reported internalization rates exceeding 80% for PELNs compared to 40% for liposomes 56. In summary, PELNs offer significant advantages over traditional liposomes across multiple critical domains, thereby presenting new avenues for future disease treatments.

PELNs, as potential drug delivery systems, can effectively transport compounds, proteins, and nucleic acids to target organs (**Table 5**).

Curcumin interacts with grape PELNs through hydrogen bonding, existing in an amorphous form within the PELNs, significantly enhancing its solubility, stability, and bioavailability 38. Hence, the interaction between active compounds and vesicles can significantly influence the overall physical and chemical properties, as well as the functionality of the entire system. Moreover, it is worthwhile to investigate whether the mode of action differs between endogenous secondary metabolites and exogenous active compounds when loaded into PELNs and their respective effects on PELNs. Apart from serving as effective delivery carriers for compounds, PELNs can also deliver nucleic acids and protect them from nuclease degradation 118, 119. Notably, orange PELNs represent a promising RNA-based vaccine delivery platform, leveraging their natural membrane envelopment to protect and deliver nucleic acids. Administering mice with orange PELNs loaded with specific mRNA via various routes generates specific IgM and IgG blocking antibodies along with T-cell immune responses, emphasizing the dual role of orange PELNs as carriers for RNA vaccines and inducers of immune responses through oral and intranasal administration 120.

**Figure 7:**
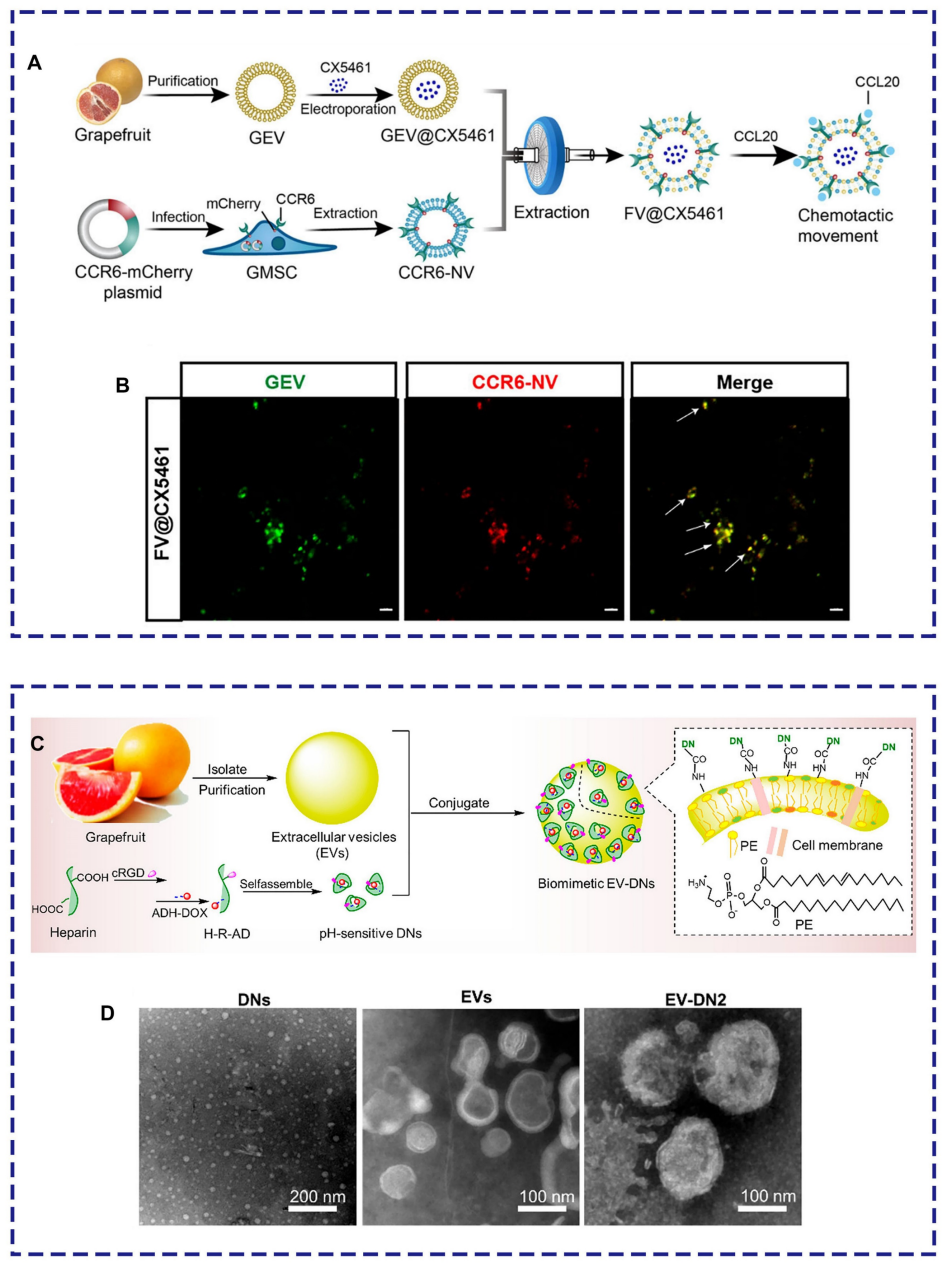
**PELNs-based biomimetic drug delivery system. (A)** Schematic illustration.** (B)** Laser confocal microscopy image demonstrating the colocalization of PELNs and CCR6-GMSCs exosomes (scale bar: 2 μm). Adapted with permission from 53, copyright 2023, Journal of Extracellular Vesicles. **(C)** Schematic illustration.** (D)** Transmission electron microscope images of DNs, grapefruit lipid-derived PELNs, and PELNs-DNs. Adapted with permission from 121, copyright 2021, Nano Letters.

Several compelling studies have focused on the modification of PELNs to improve their ability to target specific lesions. Thus, it is essential to explore various engineering approaches, including nanobiotechnology. For example, grapefruit lipid-derived PELNs loaded with the immune suppressor CX5461 fused with engineered gingiva-derived mesenchymal stem cells (GMSCs) overexpressing CCR6 selectively targeted the inflammatory area of autoimmune skin diseases upon intravenous injection, reshaping the imbalanced immune microenvironment (**Figure 7A-B**) 53. Additionally, modifying PELNs can enhance their drug-loading capacity. Niu et al. demonstrated an unprecedented four-fold increase in drug loading capacity by incorporating doxorubicin-loaded heparin nanoparticles (DNs) onto the surface of grapefruit lipid-derived PELNs using catalytic infiltration. This method effectively improved the therapeutic effectiveness against glioblastoma (**Figure 7C-D**) 121.

## 5. Toxicity of PELNs

The safety of PELNs is a crucial prerequisite for their extensive research and clinical translation. PELNs, devoid of zoonotic or human pathogens, have demonstrated favorable biocompatibility both in vitro and across various in vivo administration routes. For instance, cabbage-derived PELNs at concentrations of 1×10^11^ and 2×10^11^ particles/mL showed no significant reduction in cell viability after 72 hours of incubation in HaCaT, HDF, and Raw264.7 cells 122. To date, no toxicity reports have emerged from oral administration studies of PELNs. Daily oral administration of grapefruit PELNs (10 mg/kg) to mice for 7 days did not alter serum IFN-γ levels, liver enzymes, or AST/ALT levels 86. In addition, no significant toxicity has been observed with other administration routes, including intraperitoneal injection 110 and intranasal delivery 85.

Most PELNs intended for intravenous injection, such as those derived from grapefruit 56, ginger 66, lemon 123, and ginseng 44, have shown no evidence of potential toxicity. However, PELNs sourced from certain herbs may exhibit significant toxicity when administered intravenously. For instance, PELNs derived from tea leaves and camellia flowers have been found to induce notable hepatic and renal toxicity following intravenous injection, whereas oral administration poses no such risks 68, 69. Given the varied compositions and characteristics of PELNs derived from different sources, thorough safety assessments are indispensable prior to their potential application via intravenous administration.

## 6. Challenges in PELNs Application

PELNs have garnered significant attention for their potential therapeutic applications across different diseases. However, challenges in their application have led to a limited number of clinical trials. At present, only four clinical trials focusing on PELNs (NCT01668849, NCT04879810, NCT01294072, NCT03493984) have been undertaken, yet substantive data remain elusive despite the culmination of their primary timelines 124. This circumstance may be attributed to the ongoing status of these investigations, the deadline for results submission, or exemption of certain studies from results submission requirements. Given the current scarcity of clinical trials, it is evident that addressing these challenges and furthering clinical experiments is necessary to propel the application of PELNs within future clinical therapies.

The production of PELNs faces significant challenges, especially in isolation and content standardization. In comparison to EVs derived from cells, the isolation of PELNs is limited due to the additional preprocessing steps required for plant-derived materials. Notably, among these challenges, heterogeneity poses a critical concern in PELNs manufacturing, since EV mixtures obtained through diverse extraction methods fail to qualify unequivocally as pure exosomes. In murine models, the administration of PELNs typically involves dosages spanning from 3-10 mg/kg (protein content), equating to 77.58 mg upon extrapolation to a 60 kg adult using the body surface area methodology. Despite the abundance of sources for PELNs facilitating their mass production compared to exosomes from animal origins, refining extraction methods is essential to achieve large-scale, high-purity production of PELNs. Therefore, achieving precise PELNs extraction with identical defined contents necessitates the deployment of highly accurate tools and innovative extraction methods.

The second pivotal challenge associated with PELNs is related with the uncertainty regarding their content. Plants have diverse components and functionalities in various parts, including roots, stems, leaves, flowers, bark, fruits, seeds, and dried aerial portions. Evident differentiations in miRNA content and disease-targeting profiles exist between the underground roots and above-ground stems/leaves of houttuynia cordata-derived PELNs. Additionally, discernible dissimilarities manifest between PELNs extracted from dried and fresh plant samples. Dry plants may present greater difficulties in obtaining PELNs compared to fresh counterparts, and the cargo of PELNs derived may also exhibit variability 125. Furthermore, the cargo content of PELNs sourced from mature and immature plant samples also varies noticeably. For instance, the miRNA abundance in mature coconut-derived PELNs surpasses that in immature coconut-derived PELNs 126.

Preserving PELNs presents great challenges, particularly regarding storage conditions. Conventionally, freezing PELN samples below their reactivity threshold is a standard practice in research sample preservation. Storing ginger-derived PELNs at -80°C has proven effective in inhibiting IL-1β secretion and Casp1 self-cleavage, thereby facilitating prolonged preservation 127. However, the recurrence of freeze-thaw cycles may result in PELN aggregation. Thus, the incorporation of cryoprotectants such as glucose, sucrose, and trehalose forms a hydrating shield around PELNs during freeze-drying. While this approach holds promise for improving PELN quality during storage and transportation, its significance and effectiveness warrant further investigation 128.

The primary challenges associated with using PELNs in therapeutic applications relate to their targeting capabilities and safety. Targeting lesion areas effectively is crucial, yet it remains a significant challenge for PELNs in disease treatment. Refining their targeting capabilities through methods such as incorporating targeting ligands or cell membrane-derived vesicles can enhance their efficiency in intracellular delivery, especially in intravenous administration 129. Furthermore, it is necessary to rigorously evaluate the safety of PELNs. Although most administration routes of PELNs have shown no toxicity in current studies, concerns about safety have arisen specifically with intravenous injection in some investigations 31, 68, 69, presenting a challenge for their therapeutic application. Additionally, the safe dosages and minimum effective doses may vary across different types of PELNs. Therefore, prior to using PELNs as therapeutic agents and drug carriers, comprehensive quality control measures are imperative to address concerns regarding biological safety and potential toxicity from unidentified bioactive components. A thorough evaluation encompassing morphological characteristics, quantitative parameters, safe dosage levels and active constituents is indispensable.

## 7. Summary and outlook

In recent years, research on cell-free therapeutic approaches based on PELNs have attracted considerable attention. Plant-derived PELNs offer several advantages over exosomes derived from mammalian cells, including high safety, environmental sustainability, cost-effectiveness, and enriched biological activities (**Table 6**). As drug carriers, PELNs can exert synergistic therapeutic effects through efficient drug delivery based on their inherent biological properties combined with site-specific modifications.

At present, molecular mechanisms underlying cargo uptake and release into recipient cells remain incompletely understood. Thus, it is crucial to explore how to regulate the biogenesis of PELNs and their functions. Currently, the nomenclature of PELNs has been inconsistent in studies, which may be attributed to the differences in isolation methods as well as the particular terms such as "EVs" and "exosomes". The generally accepted size range of exosomes span from 30 nm to 200 nm. Notably, several studies have reported the size of PELNs to be more than 200 nm. For plant-derived PELNs, the plant can be named in Latin and English. What's important is to keep consistent in their nomenclature. Therefore, we should modify the protocols for the isolation and definition of PELNs and improve the clarity in terminology. Recently, a consensus statement has been issued by the Chinese Expert Committee on Research and Application of Chinese Herbal Vesicles. It involves the nomenclature, isolation methods, quality control, and applications of PELNs, providing unified recommendations for plant vesicles primarily derived from traditional Chinese herbs. With periodic updates and revisions, it was aimed to guide researchers in the field of PELNs 130.

PELNs serve as carriers for various cargo, including proteins, nucleic acids, lipids, and metabolites. As evidenced by recent studies, they are potential for therapeutic interventions. Although numerous studies have identified the components of PELNs through sequencing, elucidating the specific functions of these components should be a primary focus for future research. Secondary metabolites of PELNs have garnered significant attentions for years. In developing countries, herbal remedies usually play a predominant role in disease management. The metabolites in these herbs can regulate the functions of blood or cell-derived exosomes, thereby exerting therapeutic effects on diseases 131, 132. Thus, future research should explore the synergistic effects of PELNs and cell-derived exosomes in the treatments of diseases.

Owing to their targeting tendency for gastrointestinal tract and tumors, PELNs are primarily applied in gastrointestinal disorders, particularly colitis. Meanwhile, the significant regulatory role of PELNs in gut microbiota should not be underestimated. PELNs are primarily delivered orally, but their efficacy is limited by restricted distribution in tissues beyond the gastrointestinal tract. Engineering modifications to PELNs can effectively make them suitable for specific tissues and organs, thus addressing the challenges encountered by conventional drug delivery systems. For instance, incorporating cell membranes containing targeting ligands onto the surface of PELNs facilitates targeted delivery to specific regions, thereby achieving precise delivery of PELNs 83. PEG-modified PELNs can increase retention of PELNs in both the systemic circulation and tumor tissues, thereby further enhancing their therapeutic efficacy 16, 84. Hydrogel-loaded PELNs exhibit pseudoplastic flow behavior and viscosity suitable for local application, making them more appropriate for transdermal drug delivery 75, 133. Furthermore, incorporating drug-loaded nanocarrier structures either embedded within PELNs or patched onto their surface enhances the drug-carrying efficacy and stability of PELNs 121, 134. Although research on PELNs is still in the emerging stage compared to other engineered nanoparticles, it warrants more in-depth investigation.

Fruits and vegetables, owing to their high lipid content, serve not only biological functions but also as potential carriers for drug delivery. PELNs derived from herbs, enriched with active herbal components, exhibit diverse biological effects such as regenerative and anti-osteoporotic properties. However, research exploring their potential as drug delivery carriers is limited, leaving their role in this capacity uncertain. In addition to medicinal field, PELNs is also promising in the domain of skincare. They can facilitate the penetration of biologically active substances derived from plant cells into the skin. Besides, PELNs also have a potential in releasing encapsulated nutrients to achieve sustained nourishment. For instance, a skin-rejuvenating formulation utilizing Aloe vera-derived PELNs, which are rich in heat shock proteins, has been developed to enhance skin texture and complexion, as well as improve the appearance of scars and skin abrasions 135.

Despite the significant challenges in their production and application, the potential of PELNs in the biomedicine is immense. Over the past decade, numerous researches have been carried out for understanding the biomedical efficacy of PELNs. However, further in-depth exploration is still necessary in future. Furthermore, several promising PELNs, such as those derived from grapefruit, ginger, and ginseng, warrant further development. Grapefruit PELNs, capable of delivering nucleic acids, proteins, and small molecules, show considerable promise as drug delivery carriers. Ginger PELNs, owing to their broad therapeutic properties, represent valuable candidates for development as therapeutic agents. Ginseng PELNs have demonstrated remarkable efficacy in treating various tumors, underscoring their potential as future anti-cancer drugs. Overall, the transition of PELNs from lab to commercial production necessitates substantial endeavors, including the establishment of standardized production protocols, exploration of stable long-term storage methodologies, and the initiation of clinical trials.

## Figures and Tables

**Table 1:** Characteristics of main pre-treatment/isolation and purification methods of PELNs

	Pretreatment/separation and purification method	Advantages	Disadvantages	Main plants	Yield	Ref.
Pretreatment method	Blender juice extraction	Convenient; fast; high concentration	Cell damage may lead to degraded plasma membranes and cellular fragments	Most fruits and vegetables	/	31, 81
	Tissue infiltration homogenization	Separable nanoparticles for drying plants; high purity	Low concentration; potential cell damage; distorted plant metabolite profile	Mulberry leaf, tea leaf, panax notoginseng, lonicera japonica, sophora	/	33
Separation and purification methods	Ultracentrifugation	High sample capacity; high production	Expensive equipment; low purity; time-consuming	Grapefruit	9 × 10^10^ particles/mL juice	136
				Tomato	6 × 10^9^ particles/mL juice	137
				Orange	1 mg protein/350 mL juice	41
				Ginger	240 mg protein/kg ginger	138
				Mushroom	0.8-9.7 × 10^11^ particles/g mushroom	139
				Cabbage	0.432 × 10^9^ particles/μg protein	122
				Dendropanax morbifera	1.53 × 10^9^ particles/g leaf and 4.98 × 10^8^ particles/g stem, respectively	63
	Density gradient centrifugation	High purity; Exosome structure preserved	Low production; time-consuming	Grapefruit	2.21 ± 0.044 g/kg grapefruit	56
				Tomato	0.44 ± 0.02 g/kg tomato	56
				Grape	1.76 ± 0.15 g/kg grape	56
				Ginger	1 × 10^12^ particles/kg ginger	54
					48.5 ± 4.8 mg protein/kg ginger	66
					17.5 mg protein/kg ginger	140
				Ginseng	500 mg protein/kg ginseng	44
					168 mg protein/kg ginseng	45
					5.62 × 10^11^ particles/g ginseng	141
				Turmeric	50-100 mg protein/kg turmeric	142
	Ultrafiltration	Convenient; fast	Compromised EV structure; moderate purity	Blueberry, arabidopsis	/	143
	Size exclusion chromatography	Highly automated; the structure of PELNs remains intact; high purity	Low yield; expensive equipment	Cabbage	0.242 × 10^9^ particles/μg protein	122

EV: extracellular vesicle.

**Table 2:** Summary of the active contents in PELNs

Plant type	Plant source	Contents	Effects	Whether or not presence in humans	Ref.
Fruit	Grapefruit	Ascorbic acid	Anti-leukemic effect	No	136
	Broccoli, pomegranate, apple, orange	miR159a, miR162a, miR166b, miR396b	Toxic effect on Caco-2 cells	No	118
Vegetable	Ginger	PA	Induced Foxa2 expression in intestinal epithelial cells, interacted with HBP35	Yes	54
		aly-miR159a	Decreased the attachment of Porphyromonas gingivalis to TIGK cells	No	55
		rlcv-miR-rL1-28-3p, aly-miR396a-5p	Inhibited the expression of spike genes and Nsp3	No	58
		osa-miR-530-5p	Inhibited the synthesis of ORF1b	No	59
		mdo-miR7267-3p	Targeted the monooxygenase ycnE of LGG	No	79
	Garlic	han-miR3630-5p	Bound to the 3′ UTR of TLR4	No	144
		PA	Bound to microglial cell BASP1	Yes	100
		miR-396e	Regulated PFKFB3 expression	No	95
	Broccoli	miR5266	Bound with the 3′UTR of MMP-9	No	145
	Cucumber	Cucurbitacin B	Anti-tumor effect	No	146
Herb	Rehmanniae radix	rgl-miR-7972	Targeted GPR161 to inhibit inflammation	No	105
	Oat	β-Glucan	Bound to microglial hippocalcin	No	64
	Houttuynia cordata	miR168b-3p, miR166a-3p, miR159a	Targeted to SARS-CoV-2	No	125
	Lonicera japonica	miR2911	Targeted to E6 and E7 genes of HPV16/18	No	147
	Sophora	Rutin	Promoted nerve regeneration	No	63

BMDM: bone marrow-derived macrophage; LGG: Lactobacillus rhamnosus GG; PFKFB3: 6-phosphofructo-2-kinase/fructose-2,6-biphosphatase.

**Table 3:** Summary of common applications of PELNs derived from fruit, vegetable and herb in biotherapeutics

Biological activity	Plant type	Disease	Plant source	Main findings	Ref.
Anti-inflammatory	Fruit	Dermatitis	Apple	Downregulated NF-κB signaling pathway leads to changes in ECM production by dermal fibroblasts	148
			Grapefruit	Reduced expression of inflammatory factors and reshaping of the imbalanced immune microenvironment	53
		Colitis		Regulated inflammatory cytokine expression leading to improvement in colitis in mice	86
			Grape	Mucosal epithelial regeneration restored the entire intestinal structure in colitis mice	14
		Obesity	Orange	Ameliorated intestinal inflammation accelerated the restoration of intestinal function	41
	Vegetable	Colitis	Ginger	Regulated inflammatory cytokine expression leading to enhanced intestinal repair	20, 149
			Bitter melon	Regulated oxidative stress and inflammatory markers in the blood of mice, safeguarding the colonic mucosa	51
		Obesity	Garlic	Reduced inflammatory cytokine expression and inhibited c-Myc-mediated STING expression	100
				PFKFB3 expression in PELNs was regulated by miR-396e influencing metabolic reprogramming in macrophages	95
		Acute liver injury		Reduced macrophage infiltration via CCR2/CCR5 signaling inhibition	150
			Shiitake Mushroom	NLRP3 inflammasome activation was inhibited in macrophages	139
		Photodamage	Potato	Inhibited MMP and inflammatory cytokine expression preventing collagen degradation and promoting cell proliferation	99
	Herb	Colitis	Mulberry bark	Activated the AhR signaling pathway, mediated by HSPA8 to induce COPS8 expression	151
		Cerebral ischemia-reperfusion injury	Panax notoginseng	Induced a shift in microglial phenotypes from M1 to M2 and activated the PI3K/Akt signaling pathway	31
		Hypertension	Dandelion	Increased butyric acid production and suppressed systemic inflammation and vascular remodeling	152
		Brain inflammation	Oat	Modulated the assembly of the HPCA/Rab11a/dectin-1 complex	64
Anti-tumor	Fruit	Leukemia	Grapefruit	Inhibited leukemia cell proliferation and elevated ROS levels in leukemia progenitor cells	136
		Inflammatory tumor		Targeted delivery to tissues with inflammatory tumors	16
		Gastric cancer	Lemon	Induced ROS generation causing S-phase arrest and apoptosis in gastric cancer cells	78
		P53-inactivated colorectal cancer		Activated the macropinocytosis pathway and inhibited tumor cell proliferation	153
		Colorectal cancer		Inhibited cellular lipid metabolism and suppressed tumor cell growth	154
	Vegetable	Non-small cell lung cancer	Cucumber	Cucurbitacin B suppressed ROS generation induced by the STAT3 signaling pathway resulting in cell cycle arrest	146
		Triple-negative breast cancer	Ginger	Induced apoptosis, cell cycle arrest, and cell migration	30
		Colorectal cancer	Corn	Stimulated the release of inflammatory factors from Raw264.7 cells, and inhibited the colon26 cell proliferation	101
	Herb	Breast cancer	Tea flower/leaf	Increased ROS production leading to mitochondrial damage and cell cycle arrest	68, 69
		Hepatocellular carcinoma	Morus nigra L.	Enhanced oxidative stress caused mitochondrial damage within tumor cells and suppressed their proliferation	155
		Cervical cancer	Lonicera japonica	MiR2911 bound to HPV16/18 E6 and E7 oncogenes in PELNs inducing apoptosis	147
		Leukemia, cervical cancer	Moringa oleifera Lam	Reduced BCL2 protein expression and mitochondrial membrane potential to promote apoptosis in tumor cells	156
		Colorectal cancer	Rice bran	Induced cell cycle arrest and apoptosis, and reduced the expression of proliferative proteins	157
		Triple-negative breast cancer	Brucea javanica	Inhibited tumor growth, metastasis, and angiogenesis and activated the PI3K/Akt signaling pathway	158
		Lung cancer	Ginseng	Induced thymidine phosphorylase expression and downregulated the pentose phosphate pathway	159
		Melanoma		Induced TAM polarization from M2 to M1 phenotype through the TLR4/MyD88 pathway and increased ROS production and cell apoptosis	44
				Increased tumor-infiltrating lymphocyte infiltration, inhibited hot tumor growth and converted cold tumors into hot tumors	103
		Colorectal cancer		Reprogrammed TAMs and enhanced CD8 Teff function via the mTOR-T-bet axis and downregulated immune checkpoint expression on T cells	102
		Glioma		Promoted TAM proliferation, modulated the immune system and silenced the c-MYC gene mediated by ptc-miR396f in PELNs	45
Gut microbiota regulation	Fruit	Intestinal bacterial infection	Lemon	Inhibited the production of Msp1 and Msp3 through RNase P-mediated specific tRNA decay, and increased bile tolerance for LGG	160
				Manipulated probiotics to mediate bile resistance and intestinal survival	161
	Vegetable	Colitis	Garlic	Inhibited the TLR4/MyD88/NF-κB signaling pathway, modulated the gut microbiota, and improved tight junction protein dysfunction	144
				Bacteroides thetaiotaomicron growth was promoted by p-miR2916-p3 in PELNs	90
		Diabetes and brain inflammation	Ginger	Induced the generation of indole-3-aldehyde and IL-22, activated antimicrobial immunity and promoted tissue repair at the mucosal surface	79
			Garlic	The OMVs released by A. muciniphila were modulated, inhibiting cGas-STING-mediated inflammatory responses and crosstalk between GLP-1R and insulin pathways.	107
		Constipation	Broccoli	Restored tryptophan metabolism, ameliorated gut microbiota dysbiosis and accelerated intestinal transit	162
	Herb	Colitis	Ginseng	Balanced the gut microbiota at the intestinal barrier	67
			Portulaca oleracea L.	Promoted Lactobacillus reuteri growth and reprogrammed conventional CD4+ T cells into double-positive CD4+CD8+ T cells	163
				Modulated colonic barrier dysfunction and gut microbiota abundance	106
			Tea leaf	Enhanced the diversity and overall abundance of the gut microbiota	104
		Osteoporosis	Pueraria lobata	Promoted osteogenic differentiation and functionality of hBMSCs and increased cellular autophagy	92
		Lung inflammation	Rehmanniae radix	Activated the Hedgehog pathway and inhibited E. coli biofilm formation by targeting the virulence gene sxt2	105

ECM: extracellular matrix; LGG: Lactobacillus rhamnosus GG; MMP: matrix metalloproteinases; PFKFB3: 6-phosphofructo-2-kinase/fructose-2,6-biphosphatase 3; OMVs: outer membrane vesicles; HSPA8: heat shock protein family A member 8; COPS8: COP9 Constitutive Photomorphogenic Homolog Subunit 8; TAMs: tumor-associated macrophages; AhR: aryl hydrocarbon receptor.

**Table 4:** Summary of unique applications of PELNs derived from fruit, vegetable and herb in biotherapeutics.

Plant type	Biological activity	Disease	Plant source	Main findings	Ref.
Fruit	Antioxidant	Colitis	Lemon	Probiotics were manipulated to mediate bile resistance and enhance intestinal survival	160, 161
		Leaky gut and liver injury	Pomegranate	Reduced hepatic oxidative stress and apoptosis markers preventing intestinal leakage	111
		Non-alcoholic fatty liver disease	Blueberry	Oxidative stress and cell apoptosis were mitigated thereby ameliorating insulin resistance and hepatic dysfunction	164
		Photoaging	Golden cherry	Scavenged free radicals	133
Vegetable	Antiviral	SARS-CoV-2	Ginger	ORF1b synthesis was inhibited by targeting the ribosomal slippage site in the ORF1ab gene	59
				Spike genes and Nsp3 expression were inhibited by rlcv-miR-rL1-28-3p and aly-miR396a-5p in PELNs	58
	Regulation of insulin resistance	Insulin resistance and obesity	Ginger	Inhibited the expression of the aromatic hydrocarbon receptor improving host glucose tolerance and insulin response	91
				Foxa2 expression in intestinal epithelial cells was induced by PA in PELNs	54
Herb	Regenerative effect	Neural differentiation in vitro	Ginseng	Upregulated the PI3K signaling pathway, stimulating the differentiation and development of BMSCs	75
		Skin wound		Angiogenesis in endothelial cells was facilitated through the ERK and AKT/mTOR pathways	113
				Enhanced the angiogenesis and nascent vessel network reconstruction in full-thickness diabetic complicated skin ulcer wounds	138
			Wheat	Induced proliferation, migration, and angiogenesis	93
		Bacterial infection	Dandelion	Neutralized Staphylococcus aureus exotoxins, and promote the healing of wounds	114
		Muscle atrophy	Lycium barbarum L.	Activated the muscle regeneration and AMPK/SIRT1/PGC1α signaling pathway	165
	Anti-osteoporotic effect	Osteoporosis	Ginseng	Suppressed the IκBα, c-JUN N-terminal kinase and ERK signaling pathways and regulated genes associated with osteoclast maturation	115
			Pueraria lobata	Promoted osteogenic differentiation of hBMSCs and enhanced intracellular autophagy	92
			Yam	Activated the BMP-2/p-p38-dependent Runx2 pathway, and promoted differentiation and mineralization of osteoblasts	80

PA: phosphatidic acid; BMSCs: bone marrow mesenchymal stem cells.

**Table 5:** Summary of the applications of PELNs in drug delivery

Plant type	Plant source	Medications carried	Target cell/organ	Ref.
Fruit	Grape	Fisetin	MOLT-4 cell	166
		Curcumin	/	38
	Grapefruit	Methotrexate	Intestinal macrophage	86
		HSP70, variants of BSA	Human peripheral blood mononuclear cells, colon cancer cells	167
		HSP70	Glioma cells	137
		Doxorubicin, curcumin	Colonic tissue	16
		CX5461	Inflammatory skin tissue	53
		Doxorubicin, heparin nanoparticles	Glioma	121
		siRNA	HaCaT cell	77
		JSI-124, siRNA, paclitaxel, folic acid	Tumor	56
	Apple, orange, pomegranate	ath-miR159a, ath-miR162a-3p, ath-miR166b-3p, ath-miR396b-5p	Caco-2 cells	118
	Orange	mRNA	T cell	120
Vegetable	Celery	Doxorubicin	Tumor	168
	Sesame leaf	Luteolin	Gastrointestinal tract	169
	Allium tuberosum	Dexamethasone	Microglial cell	98
	Broccoli	ath-miR159a, ath-miR159b-3p, ath-miR166b-3p, ath-miR319a, ath-miR403-3p	Caco-2 cell	119
	Ginger	Infliximab	Gastrointestinal tract	140
		Doxorubicin, folic acid	Tumor	66
		Dmt1 siRNA	Duodenum	170
		CD98 siRNA	Colonic tissue	171
Herb	Ginseng	miR-182-5p	Colonic tissue	83

MOLT-4: human acute lymphoblastic leukemia cells; HSP70: heat shock protein 70.

**Table 6:** Comparison between PELN and mammalian cell-derived exosomes

Source of exosomes	Size	Contents	Distribution	Functionality	Advantages	Disadvantages	Ref.
PELN	30-800nm	Protein, nucleic acid, lipid, secondary metabolite	Liver, kidney, spleen, lungs, brain, stomach, intestine	Anti-inflammatory, anti-tumor, gut microbiota regulation, regenerative, drug carrier, etc.	Promising for mass production, enriched biological activities, low toxicity, high biocompatibility, and environmental-friendliness	High heterogeneity, seasonal harvest, and lack of standardized isolation methods	10, 37, 47, 129, 172, 173
Mammalian cell-derived exosomes	30-150nm	Protein, nucleic acid, lipid	Liver, kidney, spleen, lungs, brain, stomach, intestine	Anti-inflammatory, anti-tumor, antioxidant, immunomodulatory, drug carrier, etc.	High biocompatibility, noninvasive biomarker, and potential therapeutic efficacy	Complex preparation steps, low productivity, and high heterogeneity	72, 174-176

## Abbreviations

AhR: aryl hydrocarbon receptor
ARG1: arginase-1
DSS: dextran sulfate sodium
EV: extracellular vesicle
EXPO: exocyst-positive organelle
HFD: high-fat diet
IRS: insulin receptor substrates
MSC: mesenchymal stem cells
MVB: multivesicular body
OMV: outer membrane vesicle
PA: phosphatidic acid
PELN: plant-derived exosome-like nanoparticle
PEN1: penetration 1
PS: phosphatidylserine
RANKL: receptor activator of nuclear factor-kappa B ligand
SARS-CoV-2: severe acute respiratory syndrome coronavirus 2
SM: sphingomyelin
SV: small vacuole
TAM: tumor-associated macrophage
TET-8: tetraspanin-8
TME: tumor microenvironment
